# *Culex quinquefasciatus* from areas with the highest incidence of microcephaly associated with Zika virus infections in the Northeast Region of Brazil are refractory to the virus

**DOI:** 10.1590/0074-02760170145

**Published:** 2017-08

**Authors:** Rosilainy Surubi Fernandes, Stéphanie Silva Campos, Paulino Siqueira Ribeiro, Lidiane MS Raphael, Myrna C Bonaldo, Ricardo Lourenço-de-Oliveira

**Affiliations:** 1 Fundação Oswaldo Cruz, Instituto Oswaldo Cruz, Laboratório de Mosquitos Transmissores de Hematozoários, Rio de Janeiro, RJ, Brasil; 2 Fundação Oswaldo Cruz, Instituto Oswaldo Cruz, Laboratório de Biologia Molecular de Flavivírus, Rio de Janeiro, RJ, Brasil

**Keywords:** Culex quinquefasciatus, Zika virus, vector competence, Brazil

## Abstract

Zika virus (ZIKV) is widely distributed in Brazil and the Northeast Region (NE) is the most affected zone, showing the highest incidence of microcephaly associated with ZIKV congenital infections worldwide. We report attempts to infect three populations of *Culex quinquefasciatus* from severely affected sites in the NE and Southeast Region (SE) of Brazil with three strains of ZIKV isolated from these localities. An *Aedes aegypti* population from the SE was used as a positive control. All tested *Cx. quinquefasciatus* populations were refractory to the ZIKV isolates. For these reasons, we believe *Cx. quinquefasciatus* should not be considered a potential vector of ZIKV in Brazil.

After rapid expansion in the Pacific Region, the Zika virus (ZIKV) was first recognised in northeastern Brazil in 2015, followed by a countrywide epidemic that eventually spread to the entire continent (Possas et al. 2017, Zanluca et al. 2015). In 2016, 15,319 probable Zika cases were recorded in Brazil and the Northeast Region (NE) was the most affected zone, with the highest incidence (134.4/100,000 inhabitants) (portalsaude.saude.gov.br/index.php/o-inisterio/principal/secretarias/svs/boletim-epidemiologico#numerosrecentes). Moreover, the first cases of microcephaly associated with ZIKV infection were reported in this Brazilian region, which also showed the highest incidence of this condition and other congenital neurological malformations worldwide. Indeed, 76.2% of the 2,366 confirmed cases of ZIKV-associated microcephaly recorded in Brazil in 2015–2016 occurred in this region of the country (Possas et al. 2017).

The primary vector of ZIKV is *Aedes aegypti* (Ferreira-de-Brito et al. 2016, Weger-Lucarelli et al. 2016). However, due to its great abundance and anthropophilic behaviour in epidemic areas, especially in low-income districts where microcephaly was highest, *Culex quinquefasciatus* came under suspicion as an alternative ZIKV vector. Therefore, investigation of the vector competence of this species was mandatory because this knowledge could be essential to ZIKV control. To date, experimental data on *Cx. quinquefasciatus* vector competence for ZIKV have been somewhat contradictory. For instance, Guo et al. (2016) reported the detection of ZIKV RNA in the saliva of orally infected Chinese *Cx. quinquefasciatus* and claimed this species was a potential vector. In contrast, *Cx. quinquefasciatus* from Rio de Janeiro, Brazil, were shown to be unable to transmit local ZIKV isolates (Fernandes et al. 2016), a result also observed in the populations in the United State of America and Australia exposed to several ZIKV strains (Hall-Mendelin et al. 2016, Hart et al. 2017, Weger-Lucarelli et al. 2016). As vector competence is known to be geographically variable and depends on the specific combination of mosquito and virus genotypes (Lambrechts 2011, Tabachnick 2013), we challenged *Cx. quinquefasciatus* from two sites where a high incidence of microcephaly associated with ZIKV infections had been reported in NE Brazil with three Brazilian ZIKV isolates from the NE and Southeast Region (SE).

We used the F_1_ generation of *Cx. quinquefasciatus* collected from NE Brazil: Recife [state of Pernambuco (PE) (08°03’14”S 34°52’52”W)] and Campina Grande [state of Paraíba (7°13*’*51*’’*S 35°52*’*54*’’*W)]. For comparison, we used two *Cx. quinquefasciatus* populations collected from SE Brazil — Manguinhos (F_1_) and Triagem (F_>10_), districts of Rio de Janeiro, whose low vector competence previously had been determined for other ZIKV isolates (Fernandes et al. 2016) — as well as an *Ae. aegypti* colony from Urca, Rio de Janeiro (F_>10_), which previously had been shown to be a highly competent vector (Fernandes et al. 2016).

Mosquito-rearing protocols were approved by the Institutional Ethical Committee on Animal Use (CEUA-IOC license LW-34/14) at the Oswaldo Cruz Institute, Oswaldo Cruz Foundation. No specific permits were required to collect mosquitoes in the districts in Recife, Campina Grande and Rio de Janeiro.

All ZIKV strains used belong to the Asian lineage and were previously isolated from humans: ZIKVPE243, from the city of Recife, PE, NE (Donald et al. 2016); ZIKVSPH2015 from the city of Sumaré, state of São Paulo, SE (Faria et al. 2016); and ZIKV RioU-1, from Rio de Janeiro, SE (Bonaldo et al. 2016). ZIKVSPH2015 has high similarity with ZIKVPE243 (99.9% of the nucleotides and 99.97% of the aminoacids) (Donald et al. 2016).

Female mosquitoes at five–seven days post-emergence were fed using an artificial feeding apparatus with a mixture containing two parts washed erythrocytes and one part viral suspension. Depending on the availability, mosquitoes were examined at seven, 14 and 21 days post-oral challenge (dpi). Homogenates of the body (thorax + abdomen) and head were examined by plaque assays in a culture of Vero cells to determine the infection (IR) and dissemination (DR) rates, respectively. We performed a real-time quantitative polymerase chain reaction (RT-qPCR) to confirm positivity for the *Culex* body samples (for details, see Fernandes et al. 2016). Saliva was also collected and stored at -80°C for further examination if evidence existed of viral dissemination.

All tested *Cx. quinquefasciatus* were refractory to ZIKV regardless of the viral strain (Table). Only one of 20 bodies of *Cx. quinquefasciatus* from Recife challenged with the ZIKV Rio-U1 was feebly positive at 7 dpi and the virus did not disseminate in this individual, as shown by the head repeatedly testing negative. As the virus did not disseminate in any *Cx. quinquefasciatus*, the saliva was not examined.

**TABLE:** Infection (IR) and dissemination (DIR) rates of Brazilian *Culex quinquefasciatus* challenged with three Zika virus (ZIKV) isolates at seven, 14, and 21 days after oral exposure

Virus solate/ origin/year/ titer (PFU/mL)	Mosquito population	Days after exposure	Number examined^*a*^ (per time point)	IR^*a*^ (per time point)	DIR^*a*^ (per time point)
ZIKVPE243/ Recife, NE/ 2015/ 2.3 x 10^6^	*Cx. quinquefasciatus* Recife, NE (F_1_) *Cx. quinquefasciatus* Campina Grande, NE (F_1_) *Cx. quinquefasciatus* Rio de Janeiro, SE (F_>10_) *Cx. quinquefasciatus* Rio de Janeiro, SE (F_1_)	7, 14 7, 14 7, 14 7, 14	20, 20 20, 20 30, 30 30, 30	0, 0 0, 0 0, 0 0, 0	- - - -
	*Aedes aegypti* Rio de Janeiro^*b*^, SE (F_>10_)	7, 14	20, 39	65%, 68%	30%, 86%
ZIKVSPH/ Sumaré, SE/ 2015/ 1.68 x 10^7^	*Cx. quinquefasciatus* Recife, NE (F_1_) *Cx. quinquefasciatus* Campina Grande, NE (F_1_) *Cx. quinquefasciatus* Rio de Janeiro, SE (F_>10_) *Cx. quinquefasciatus* Rio de Janeiro, SE (F_1_)	7, 14 7, 14 14, 21 14, 21	12, 20 20, 20 30, 3 30, 8	0, 0 0, 0 0, 0 0, 0	- - - -
	*Ae. aegypti* Rio de Janeiro^*b*^, SE (F_>10_)	14	20	100%	100%
ZIKVU1/ Rio de Janeiro, SE/ 2015/ 3.55 x 10^6^	*Cx. quinquefasciatus* Recife, NE (F_1_) *Cx. quinquefasciatus* Campina Grande, NE (F_1_)	7, 14 7, 14	20, 20 20, 20	5%, 0% 0, 0	0%, 0% -
	*Ae. aegypti* Rio de Janeiro^*b*^, SE (F_>10_)	7	20	75%	60%

*a*: the number of examined mosquitoes and infection and dissemination rates at each day post-ZIKV exposure are respectively given; *b:* Urca population; NE: Northeast Region; PFU: plaque-forming unit; SE: Southeast Region.

In contrast, all strains of ZIKV infected and disseminated in *Ae. aegypt*, regardless of the geographical origin of the isolates. IRs in *Ae. aegypti* ranged from 65–75% at 7 dpi and from 68–100% at 14 dpi; the DR ranged from 86–100% at 14 dpi. The Urca *Ae. aegypti* population had previously exhibited high transmission rates (saliva infection) to two local ZIKV (Fernandes et al. 2016) and, thus, the saliva of the infected individuals was not examined.

This is the first time that populations of *Cx. quinquefasciatus* from an area with a high incidence of microcephaly and other congenital malformations associated with ZIKV infections have been tested for vector competence to ZIKV from the same region. In agreement with results from SE Brazilian populations of *Cx. quinquefasciatus*, the tested populations were not competent for transmitting the virus, including a strain isolated from the same epidemiological region. Our results showing the refractoriness of these populations to ZIKV are consistent with studies on this and other members of the *Culex pipiens* complex (Amraoui et al. 2016, Fernandes et al. 2016, Hall-Mendelin et al. 2016, Hart et al. 2017, Weger-Lucarelli et al. 2016). The only exception reported in the literature is from Guo et al. (2016), who reported the detection of ZIKV RNA in the bodies and saliva of orally challenged Chinese *Cx. quinquefasciatus* mosquitoes*.* The detection of residual RNA, cross-reactions and cross-contamination with positive control material or other problems with molecular assays without adequate negative controls may explain this isolated discrepant result. Indeed, another Chinese *Cx. quinquefasciatus* orally exposed to a ZIKV isolate from China was not competent to transmit the virus (Liu et al. 2017). In addition, *Cx. quinquefasciatus* mosquitoes from several other geographical origins and epidemiological situations consistently have been shown to be refractory to ZIKV for both the African and Asian genotypes, even when challenged with blood meals with high viral titres (Fernandes et al. 2016, Hall-Mendelin et al. 2016, Hart et al. 2017).

Thus, evidences from the current study and from previously published works reinforce the conclusion that *Cx. quinquefasciatus* should not be considered a potential vector of ZIKV. Even when the mosquito and ZIKV isolates are from localities with a high incidence of human cases of Zika, *Cx. quinquefasciatus* is still not competent in the laboratory as a vector.
